# Metaplastic Carcinoma of the Breast with Squamous Differentiation: A Case Report from the University Teaching Hospital of Kigali (CHUK), Rwanda

**DOI:** 10.1155/2020/4806342

**Published:** 2020-08-29

**Authors:** Delphine Uwamariya, Carine Nyampinga, Anne Yvette Nsenguwera, Belson Rugwizangoga

**Affiliations:** ^1^Department of Clinical Biology, School of Medicine and Pharmacy, University of Rwanda, Kigali, Rwanda; ^2^Department of Pathology, University Teaching Hospital of Kigali, Kigali, Rwanda

## Abstract

Metaplastic breast carcinoma is a rare and aggressive condition, accounting less than 1% of breast malignancies. It presents with large mass and frequently with distant metastasis at time of diagnosis. Morphologically, it is characterized by the differentiation of neoplastic epithelium into epithelial or mesenchymal-looking elements like squamous cells, spindle cells, cartilage, or bone and has poor prognosis with its triple negative status.

## 1. Introduction

Metaplastic carcinoma of the breast is a rare condition that accounts for less than 1% of breast malignancies. They are aggressive tumors that have a worse prognosis than other triple negative invasive ductal carcinomas [1, 2]. Metaplastic carcinomas of the breast are characterized by large tumor size and rapid growth and have a high potential for metastatic spread to the lung and bone via vasculature rather than by way of lymphatics [3]. Morphologically, they are characterized by neoplastic epithelial or mesenchymal differentiation. Of the epithelial variant, the squamous cell carcinoma is the most common. Those with mesenchymal differentiation can present with proliferation of spindle cells, cartilage, or bone. Metaplastic carcinomas frequently present as well-demarcated tumors and therefore have many similarities with some variants of invasive ductal carcinoma and also with benign lesions on mammography [3]. Treatment for metaplastic breast carcinoma is relatively unknown because of the rarity of the disease, but studies suggest that the removal of the tumor and adjuvant radiation therapy has the greatest impact [3]. In this paper, we report a case of squamous metaplastic carcinoma, diagnosed in Rwanda.

## 2. Case Report

A 39-year old female patient had a 2-year history of left breast mass that measured 4 × 3 × 2.5 cm by clinical examination; imaging information is not available. An attempt to excise the mass was done at the district hospital; the histopathological examination reported it to be a poorly differentiated ductal carcinoma with cystic degeneration (immunohistochemistry results and information on the margin status are not available). The tumor recurred 6 months after the initial surgery. At this point, she was referred to a tertiary hospital where she underwent modified radical mastectomy; the specimen was submitted for histopathology examination.

On gross examination, the mastectomy weighed 300 g and measured 16 × 16 × 7 cm. The axillary tail measured 8 cm in length. The ellipsoid skin flap measured 12 × 9 cm and demonstrated an everted, grossly unremarkable nipple. On serial sections, the specimen shows a cystic lesion (3.5 cm in the greatest dimension) in the lower inner quadrant of the breast. The cystic cavity was filled with hemorrhagic fluid and necrotic debris. Grossly, the tumor was at 0.2 cm from the deep margin and 1.1 cm from the anterior-inferior margin. Nineteen lymph nodes were isolated from the axillary dissection and were all submitted for microscopic examination.

**Figure 1:**
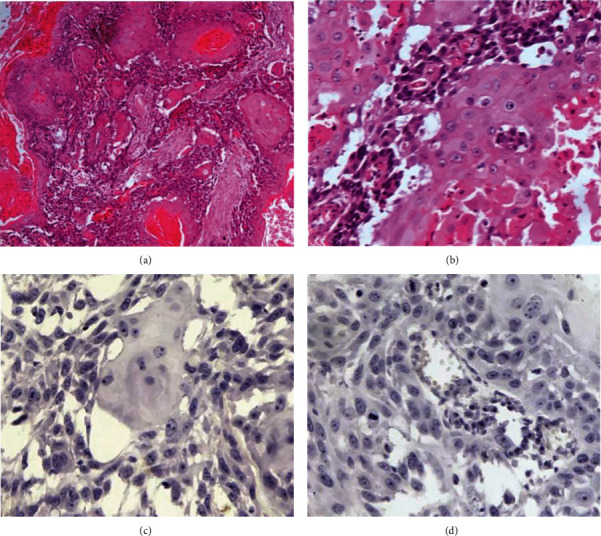
Photomicrographs of a breast metaplastic carcinoma. (a) Low magnification showing a solid trabecular tumor exhibiting keratin pearls and infiltrating the desmoplastic stroma (H&E, x40). (b) High magnification shows nests of cohesive, large polygonal cells with eosinophilic cytoplasm, and large nuclei with prominent nucleoli; necrosis and cavitation are seen on the right lower field (H&E, x200). (c) The tumor is negative for ER (immunoperoxidase, x200). (d) The tumor is negative for HER2/neu (immunoperoxidase, x200).

Microscopically, there was a 3.5 cm infiltrating tumor showing both cystic and solid areas. The solid component showed squamous nests with keratinization, invading in desmoplastic stroma (Figure 1(a)). The tumor cells are large polygonal, with eosinophilic cytoplasm, large irregular nuclei, some of which showed prominent nucleoli (Figure 1(b)). The tumor was characterized by prominent mitotic activity and lack of tubule/gland formation. Vascular invasion was present. No in situ lesions (ductal carcinoma in situ or lobular carcinoma in situ) were identified in the specimen. All nineteen lymph nodes submitted for evaluation were negative for malignancy (0/19). Immunoperoxidase stains were negative for estrogen receptor (ER, Figure 1(c)) and HER2/neu (Figure 1(d)). The final diagnosis was breast metaplastic carcinoma of squamous type, with pathologic prognostic stage IIA. The patient did not receive chemotherapy or radiation therapy; she is doing well with no evidence of local or systemic recurrence after a period of 2 years.

## 3. Discussion

Breast metaplastic carcinoma frequently presents with a palpable mass mainly in women older than 50 years of age [2, 3]. The present case is from a 39-year old female, with a 3.5 cm tumor. Metaplastic carcinomas are an uncommon heterogeneous group of tumors characterized by the histologic presence of two or more cellular elements, commonly a mixture of epithelial (i.e., squamous) and mesenchymal (i.e., chondroid and osseous) differentiation; this entity represents 0.25–1% of breast cancers diagnosed annually [4]. This mixed cell differentiation is seen both morphologically and immunophenotypically, as evidenced by immunohistochemical expression of markers of mesenchymal cells (vimentin), epithelial cells (pancytokeratin), and myoepithelial cells (S-100, smooth-muscle actin, and p63) [5].

Metaplastic carcinoma of the breast is currently classified in six subtypes, namely, (i) low-grade adenosquamous carcinoma, (ii) fibromatosis-like metaplastic carcinoma, (iii) spindle-cell carcinoma, (iv) squamous cell carcinoma, (v) metaplastic carcinoma with heterologous mesenchymal differentiation, and (vi) mixed metaplastic carcinoma [6].

The prognosis of patients with MBC depends on its grading and staging and also varies with the subtypes where fibromatosis-like carcinomas and low-grade adenosquamous carcinomas show more indolent behavior, while high-grade spindle cell, squamous cell, adenosquamous, and mixed metaplastic carcinoma are associated with the worst prognosis, matrix-producing carcinomas being associated with good prognosis [6, 7].

MBC has a lower rate of axillary lymph node metastasis compared to the invasive breast carcinoma of the same size, and late distant metastasis can be documented without lymph node involvement [3].

It has been shown that metaplastic carcinoma of the breast does not respond well to hormonal and chemotherapy treatment as other triple negative invasive breast carcinomas and that neoadjuvant radiation therapy combined with surgery can ameliorate the outcome of the patient [8].

## 4. Conclusion

In conclusion, the rarity, aggressiveness, and triple negativity of metaplastic carcinoma of the breast are important factors that influence the outcome of the patient. Early diagnosis and wide local excision of the squamous type of breast metaplastic carcinoma mass may warrant a good prognosis, as reported in this case.
